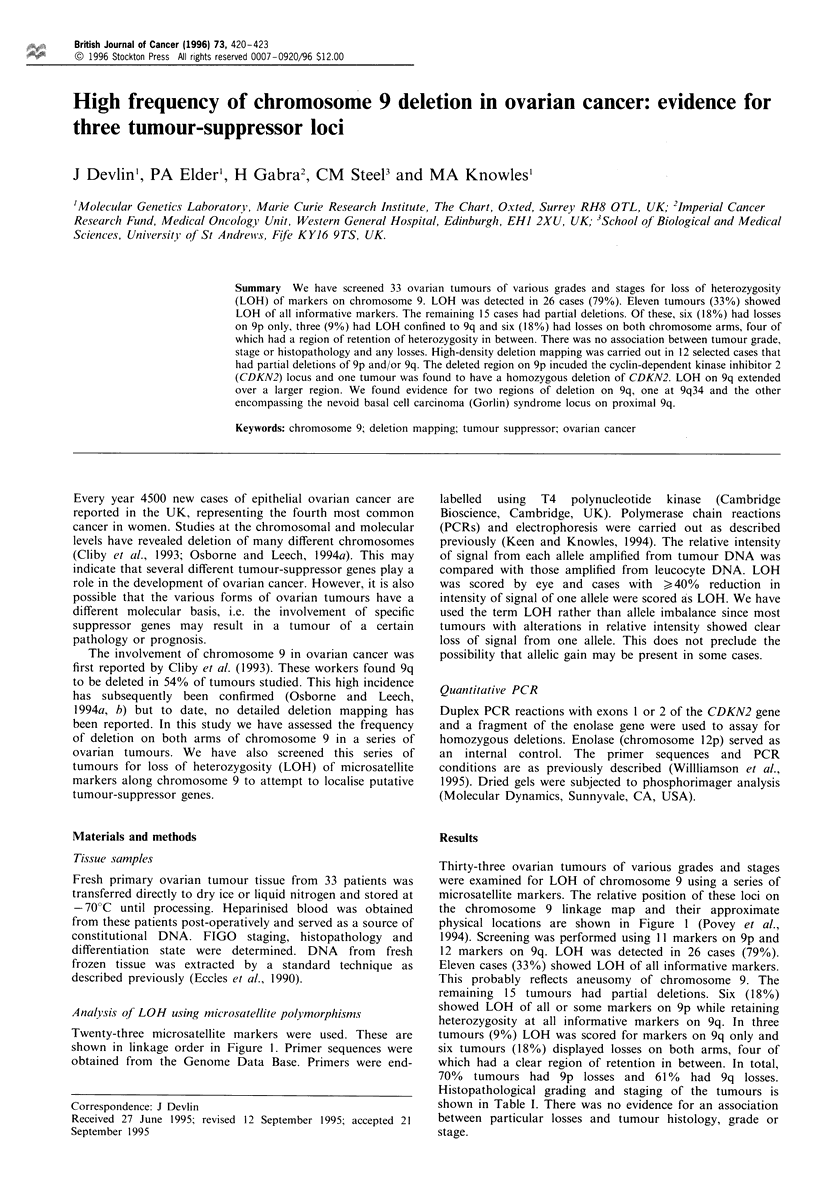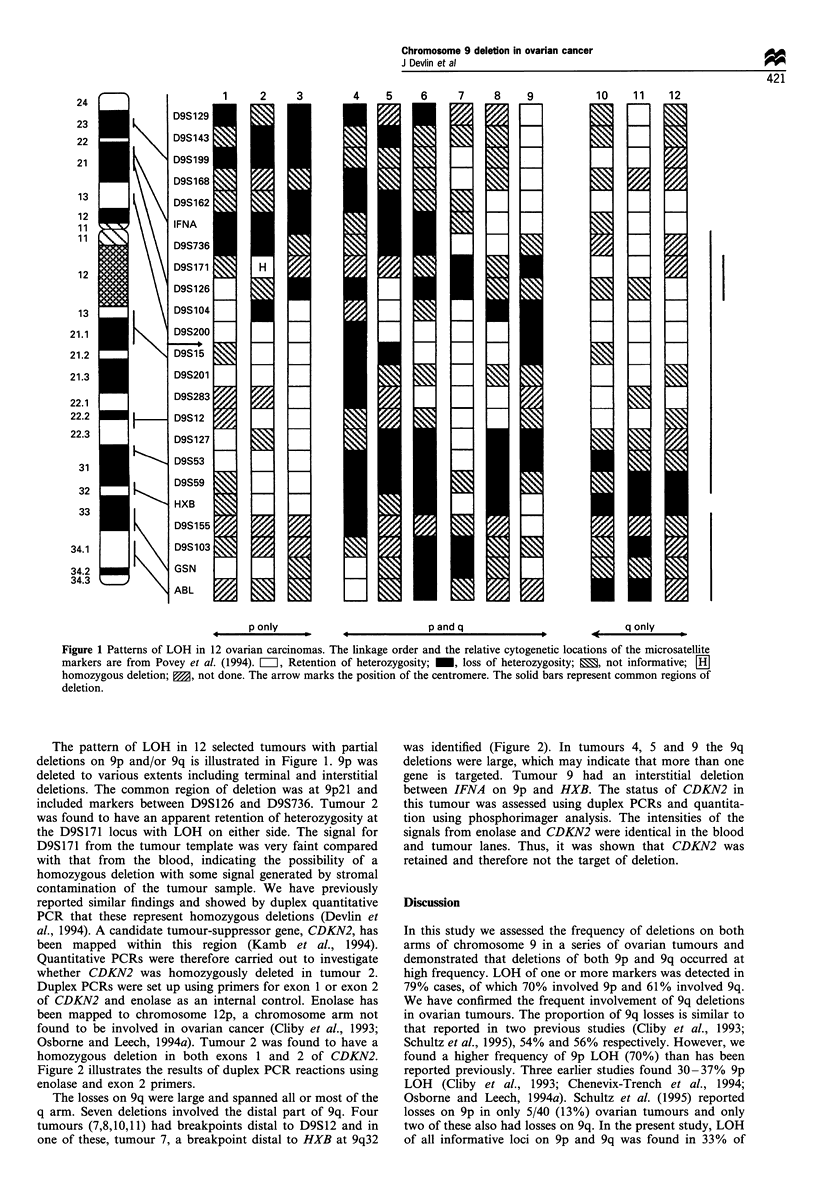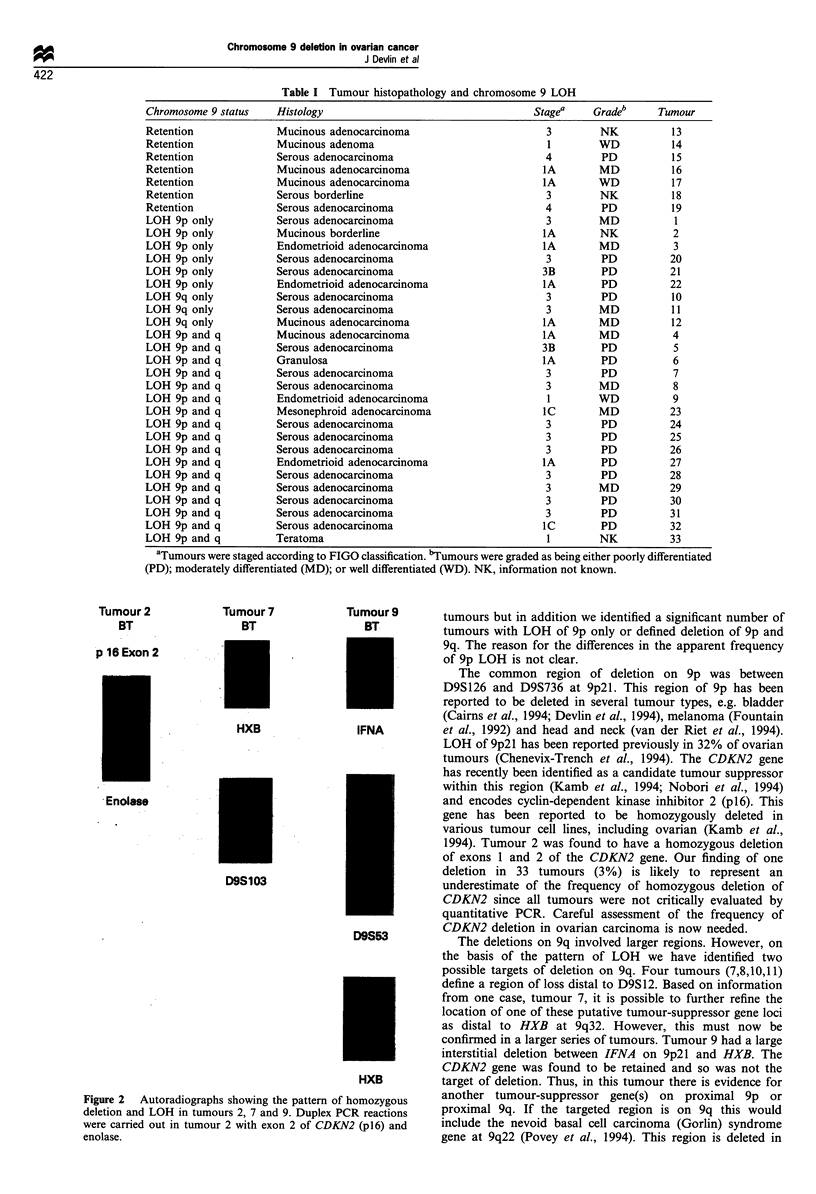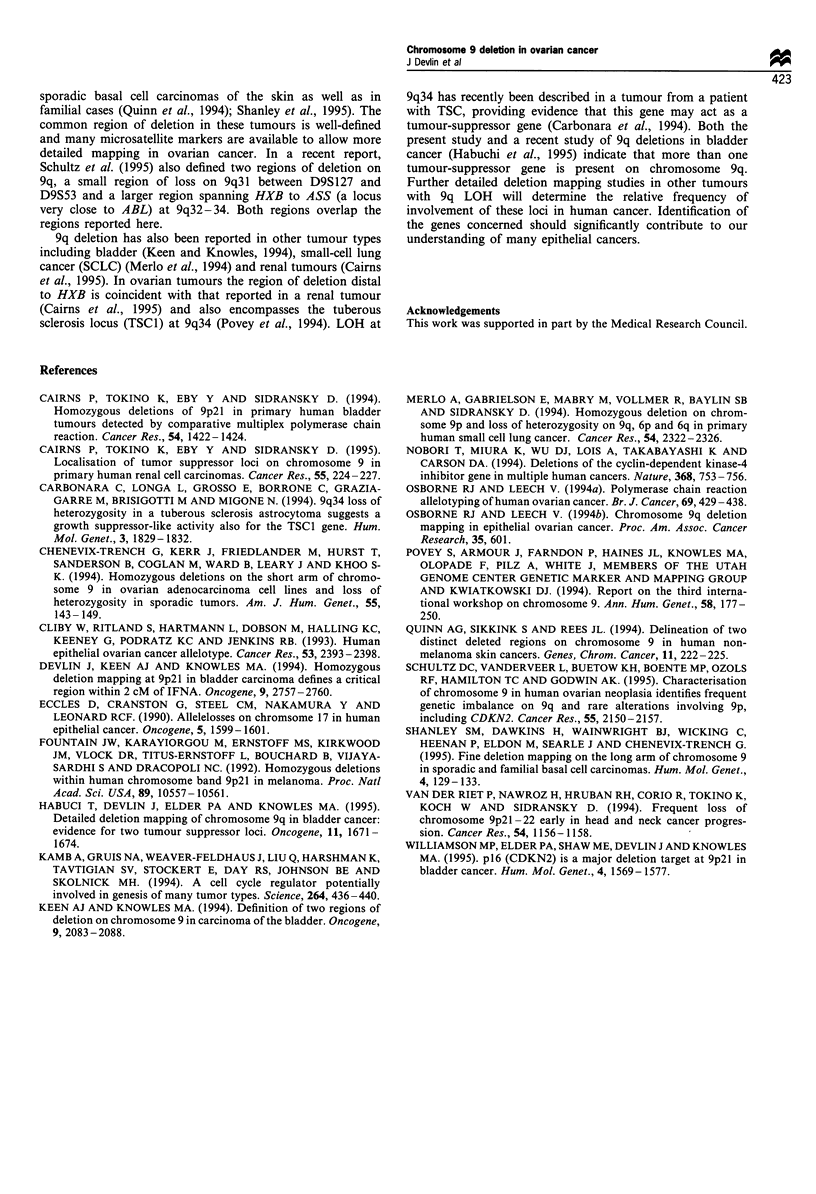# High frequency of chromosome 9 deletion in ovarian cancer: evidence for three tumour-suppressor loci.

**DOI:** 10.1038/bjc.1996.75

**Published:** 1996-02

**Authors:** J. Devlin, P. A. Elder, H. Gabra, C. M. Steel, M. A. Knowles

**Affiliations:** Molecular Genetics Laboratory, Marie Curie Institute, Oxted, Surrey, UK.

## Abstract

**Images:**


**British Journal of Cancer (1996) 73,** 420–423
© 1996 Stockton Press All rights reserved 0007-0920/96 $12.00

### High frequency of chromosome 9 deletion in ovarian cancer: evidence for three tumour-suppressor loci

J Devlin[1], PA Elder[1], H Gabra[2], CM Steel[3] and MA Knowles[1]

*[1]Molecular Genetics Laboratory, Marie Curie Research Institute, The Chart, Oxted, Surrey RH8 OTL, UK; [2]Imperial Cancer Research Fund, Medical Oncology Unit, Western General Hospital, Edinburgh, EH1 2XU, UK; [3]School of Biological and Medical Sciences, University of St Andrews, Fife KY16 9TS, UK.*

**Summary** We have screened 33 ovarian tumours of various grades and stages for loss of heterozygosity (LOH) of markers on chromosome 9. LOH was detected in 26 cases (79%). Eleven tumours (33%) showed LOH of all informative markers. The remaining 15 cases had partial deletions. Of these, six (18%) had losses on 9p only, three (9%) had LOH confined to 9q and six (18%) had losses on both chromosome arms, four of which had a region of retention of heterozygosity in between. There was no association between tumour grade, stage or histopathology and any losses. High-density deletion mapping was carried out in 12 selected cases that had partial deletions of 9p and/or 9q. The deleted region on 9p incuded the cyclin-dependent kinase inhibitor 2 (*CDKN2*) locus and one tumour was found to have a homozygous deletion of *CDKN2*. LOH on 9q extended over a larger region. We found evidence for two regions of deletion on 9q, one at 9q34 and the other encompassing the nevoid basal cell carcinoma (Gorlin) syndrome locus on proximal 9q.

**Keywords:** chromosome 9; deletion mapping; tumour suppressor; ovarian cancer

Every year 4500 new cases of epithelial ovarian cancer are reported in the UK, representing the fourth most common cancer in women. Studies at the chromosomal and molecular levels have revealed deletion of many different chromosomes (Cliby *et al.*, 1993; Osborne and Leech, 1994*a*). This may indicate that several different tumour-suppressor genes play a role in the development of ovarian cancer. However, it is also possible that the various forms of ovarian tumours have a different molecular basis, i.e. the involvement of specific suppressor genes may result in a tumour of a certain pathology or prognosis.

The involvement of chromosome 9 in ovarian cancer was first reported by Cliby *et al.* (1993). These workers found 9q to be deleted in 54% of tumours studied. This high incidence has subsequently been confirmed (Osborne and Leech, 1994*a*, *b*) but to date, no detailed deletion mapping has been reported. In this study we have assessed the frequency of deletion on both arms of chromosome 9 in a series of ovarian tumours. We have also screened this series of tumours for loss of heterozygosity (LOH) of microsatellite markers along chromosome 9 to attempt to localise putative tumour-suppressor genes.

### Materials and methods

### *Tissue samples*

Fresh primary ovarian tumour tissue from 33 patients was transferred directly to dry ice or liquid nitrogen and stored at −70°C until processing. Heparinised blood was obtained from these patients post-operatively and served as a source of constitutional DNA. FIGO staging, histopathology and differentiation state were determined. DNA from fresh frozen tissue was extracted by a standard technique as described previously (Eccles *et al.*, 1990).

### *Analysis of LOH using microsatellite polymorphisms*

Twenty-three microsatellite markers were used. These are shown in linkage order in Figure 1. Primer sequences were obtained from the Genome Data Base. Primers were end-labelled using T4 polynucleotide kinase (Cambridge Bioscience, Cambridge, UK). Polymerase chain reactions (PCRs) and electrophoresis were carried out as described previously (Keen and Knowles, 1994). The relative intensity of signal from each allele amplified from tumour DNA was compared with those amplified from leucocyte DNA. LOH was scored by eye and cases with ⩾40% reduction in intensity of signal of one allele were scored as LOH. We have used the term LOH rather than allele imbalance since most tumours with alterations in relative intensity showed clear loss of signal from one allele. This does not preclude the possibility that allelic gain may be present in some cases.

### *Quantitative PCR*

Duplex PCR reactions with exons 1 or 2 of the *CDKN2* gene and a fragment of the enolase gene were used to assay for homozygous deletions. Enolase (chromosome 12p) served as an internal control. The primer sequences and PCR conditions are as previously described (Willliamson *et al.*, 1995). Dried gels were subjected to phosphorimager analysis (Molecular Dynamics, Sunnyvale, CA, USA).

### Results

Thirty-three ovarian tumours of various grades and stages were examined for LOH of chromosome 9 using a series of microsatellite markers. The relative position of these loci on the chromosome 9 linkage map and their approximate physical locations are shown in Figure 1 (Povey *et al.*, 1994). Screening was performed using 11 markers on 9p and 12 markers on 9q. LOH was detected in 26 cases (79%). Eleven cases (33%) showed LOH of all informative markers. This probably reflects aneusomy of chromosome 9. The remaining 15 tumours had partial deletions. Six (18%) showed LOH of all or some markers on 9p while retaining heterozygosity at all informative markers on 9q. In three tumours (9%) LOH was scored for markers on 9q only and six tumours (18%) displayed losses on both arms, four of which had a clear region of retention in between. In total, 70% tumours had 9p losses and 61% had 9q losses. Histopathological grading and staging of the tumours is shown in Table I. There was no evidence for an association between particular losses and tumour histology, grade or stage.

Correspondence: J Devlin
Received 27 June 1995; revised 12 September 1995; accepted 21 September 1995

**Figure 1** Patterns of LOH in 12 ovarian carcinomas. The linkage order and the relative cytogenetic locations of the microsatellite markers are from Povey *et al.* (1994). ▭, Retention of heterozygosity; ■, loss of heterozygosity; ▨, not informative; [H] homozygous deletion; ▨, not done. The arrow marks the position of the centromere. The solid bars represent common regions of deletion.

The pattern of LOH in 12 selected tumours with partial deletions on 9p and/or 9q is illustrated in Figure 1. 9p was deleted to various extents including terminal and interstitial deletions. The common region of deletion was at 9p21 and included markers between D9S126 and D9S736. Tumour 2 was found to have an apparent retention of heterozygosity at the D9S171 locus with LOH on either side. The signal for D9S171 from the tumour template was very faint compared with that from the blood, indicating the possibility of a homozygous deletion with some signal generated by stromal contamination of the tumour sample. We have previously reported similar findings and showed by duplex quantitative PCR that these represent homozygous deletions (Devlin *et al.*, 1994). A candidate tumour-suppressor gene, *CDKN2*, has been mapped within this region (Kamb *et al.*, 1994). Quantitative PCRs were therefore carried out to investigate whether *CDKN2* was homozygously deleted in tumour 2. Duplex PCRs were set up using primers for exon 1 or exon 2 of *CDKN2* and enolase as an internal control. Enolase has been mapped to chromosome 12p, a chromosome arm not found to be involved in ovarian cancer (Cliby *et al.*, 1993; Osborne and Leech, 1994*a*). Tumour 2 was found to have a homozygous deletion in both exons 1 and 2 of *CDKN2*. Figure 2 illustrates the results of duplex PCR reactions using enolase and exon 2 primers.

The losses on 9q were large and spanned all or most of the q arm. Seven deletions involved the distal part of 9q. Four tumours (7,8,10,11) had breakpoints distal to D9S12 and in one of these, tumour 7, a breakpoint distal to *HXB* at 9q32 was identified (Figure 2). In tumours 4, 5 and 9 the 9q deletions were large, which may indicate that more than one gene is targeted. Tumour 9 had an interstitial deletion between *IFNA* on 9p and *HXB*. The status of *CDKN2* in this tumour was assessed using duplex PCRs and quantitation using phosphorimager analysis. The intensities of the signals from enolase and *CDKN2* were identical in the blood and tumour lanes. Thus, it was shown that *CDKN2* was retained and therefore not the target of deletion.

### Discussion

In this study we assessed the frequency of deletions on both arms of chromosome 9 in a series of ovarian tumours and demonstrated that deletions of both 9p and 9q occurred at high frequency. LOH of one or more markers was detected in 79% cases, of which 70% involved 9p and 61% involved 9q. We have confirmed the frequent involvement of 9q deletions in ovarian tumours. The proportion of 9q losses is similar to that reported in two previous studies (Cliby *et al.*, 1993; Schultz *et al.*, 1995), 54% and 56% respectively. However, we found a higher frequency of 9p LOH (70%) than has been reported previously. Three earlier studies found 30–37% 9p LOH (Cliby *et al.*, 1993; Chenevix-Trench *et al.*, 1994; Osborne and Leech, 1994*a*). Schultz *et al.* (1995) reported losses on 9p in only 5/40 (13%) ovarian tumours and only two of these also had losses on 9q. In the present study, LOH of all informative loci on 9p and 9q was found in 33% of

**Table I** Tumour histopathology and chromosome 9 LOH

| *Chromosome 9 status* | *Histology* | *Stage*[a] | *Grade*[b] | *Tumour* |
|---|---|---|---|---|
| Retention | Mucinous adenocarcinoma | 3 | NK | 13 |
| Retention | Mucinous adenoma | 1 | WD | 14 |
| Retention | Serous adenocarcinoma | 4 | PD | 15 |
| Retention | Mucinous adenocarcinoma | 1A | MD | 16 |
| Retention | Mucinous adenocarcinoma | 1A | WD | 17 |
| Retention | Serous borderline | 3 | NK | 18 |
| Retention | Serous adenocarcinoma | 4 | PD | 19 |
| LOH 9p only | Serous adenocarcinoma | 3 | MD | 1 |
| LOH 9p only | Mucinous borderline | 1A | NK | 2 |
| LOH 9p only | Endometrioid adenocarcinoma | 1A | MD | 3 |
| LOH 9p only | Serous adenocarcinoma | 3 | PD | 20 |
| LOH 9p only | Serous adenocarcinoma | 3B | PD | 21 |
| LOH 9p only | Endometrioid adenocarcinoma | 1A | PD | 22 |
| LOH 9q only | Serous adenocarcinoma | 3 | PD | 10 |
| LOH 9q only | Serous adenocarcinoma | 3 | MD | 11 |
| LOH 9q only | Mucinous adenocarcinoma | 1A | MD | 12 |
| LOH 9p and q | Mucinous adenocarcinoma | 1A | MD | 4 |
| LOH 9p and q | Serous adenocarcinoma | 3B | PD | 5 |
| LOH 9p and q | Granulosa | 1A | PD | 6 |
| LOH 9p and q | Serous adenocarcinoma | 3 | PD | 7 |
| LOH 9p and q | Serous adenocarcinoma | 3 | MD | 8 |
| LOH 9p and q | Endometrioid adenocarcinoma | 1 | WD | 9 |
| LOH 9p and q | Mesonephroid adenocarcinoma | 1C | MD | 23 |
| LOH 9p and q | Serous adenocarcinoma | 3 | PD | 24 |
| LOH 9p and q | Serous adenocarcinoma | 3 | PD | 25 |
| LOH 9p and q | Serous adenocarcinoma | 3 | PD | 26 |
| LOH 9p and q | Endometrioid adenocarcinoma | 1A | PD | 27 |
| LOH 9p and q | Serous adenocarcinoma | 3 | PD | 28 |
| LOH 9p and q | Serous adenocarcinoma | 3 | MD | 29 |
| LOH 9p and q | Serous adenocarcinoma | 3 | PD | 30 |
| LOH 9p and q | Serous adenocarcinoma | 3 | PD | 31 |
| LOH 9p and q | Serous adenocarcinoma | 1C | PD | 32 |
| LOH 9p and q | Teratoma | 1 | NK | 33 |

[a]Tumours were staged according to FIGO classification. [b]Tumours were graded as being either poorly differentiated (PD); moderately differentiated (MD); or well differentiated (WD). NK, information not known.

**Figure 2** Autoradiographs showing the pattern of homozygous deletion and LOH in tumours 2, 7 and 9. Duplex PCR reactions were carried out in tumour 2 with exon 2 of *CDKN2* (p16) and enolase.

tumours but in addition we identified a significant number of tumours with LOH of 9p only or defined deletion of 9p and 9q. The reason for the differences in the apparent frequency of 9p LOH is not clear.

The common region of deletion on 9p was between D9S126 and D9S736 at 9p21. This region of 9p has been reported to be deleted in several tumour types, e.g. bladder (Cairns *et al.*, 1994; Devlin *et al.*, 1994), melanoma (Fountain *et al.*, 1992) and head and neck (van der Riet *et al.*, 1994). LOH of 9p21 has been reported previously in 32% of ovarian tumours (Chenevix-Trench *et al.*, 1994). The *CDKN2* gene has recently been identified as a candidate tumour suppressor within this region (Kamb *et al.*, 1994; Nobori *et al.*, 1994) and encodes cyclin-dependent kinase inhibitor 2 (p16). This gene has been reported to be homozygously deleted in various tumour cell lines, including ovarian (Kamb *et al.*, 1994). Tumour 2 was found to have a homozygous deletion of exons 1 and 2 of the *CDKN2* gene. Our finding of one deletion in 33 tumours (3%) is likely to represent an underestimate of the frequency of homozygous deletion of *CDKN2* since all tumours were not critically evaluated by quantitative PCR. Careful assessment of the frequency of *CDKN2* deletion in ovarian carcinoma is now needed.

The deletions on 9q involved larger regions. However, on the basis of the pattern of LOH we have identified two possible targets of deletion on 9q. Four tumours (7,8,10,11) define a region of loss distal to D9S12. Based on information from one case, tumour 7, it is possible to further refine the location of one of these putative tumour-suppressor gene loci as distal to *HXB* at 9q32. However, this must now be confirmed in a larger series of tumours. Tumour 9 had a large interstitial deletion between *IFNA* on 9p21 and *HXB*. The *CDKN2* gene was found to be retained and so was not the target of deletion. Thus, in this tumour there is evidence for another tumour-suppressor gene(s) on proximal 9p or proximal 9q. If the targeted region is on 9q this would include the nevoid basal cell carcinoma (Gorlin) syndrome gene at 9q22 (Povey *et al.*, 1994). This region is deleted in sporadic basal cell carcinomas of the skin as well as in familial cases (Quinn *et al.*, 1994); Shanley *et al.*, 1995). The common region of deletion in these tumours is well-defined and many microsatellite markers are available to allow more detailed mapping in ovarian cancer. In a recent report, Schultz *et al.* (1995) also defined two regions of deletion on 9q, a small region of loss on 9q31 between D9S127 and D9S53 and a larger region spanning *HXB* to *ASS* (a locus very close to *ABL*) at 9q32–34. Both regions overlap the regions reported here.

9q deletion has also been reported in other tumour types including bladder (Keen and Knowles, 1994), small-cell lung cancer (SCLC) (Merlo *et al.*, 1994) and renal tumours (Cairns *et al.*, 1995). In ovarian tumours the region of deletion distal to *HXB* is coincident with that reported in a renal tumour (Cairns *et al.*, 1995) and also encompasses the tuberous sclerosis locus (TSC1) at 9q34 (Povey *et al.*, 1994). LOH at 9q34 has recently been described in a tumour from a patient with TSC, providing evidence that this gene may act as a tumour-suppressor gene (Carbonara *et al.*, 1994). Both the present study and a recent study of 9q deletions in bladder cancer (Habuchi *et al.*, 1995) indicate that more than one tumour-suppressor gene is present on chromosome 9q. Further detailed deletion mapping studies in other tumours with 9q LOH will determine the relative frequency of involvement of these loci in human cancer. Identification of the genes concerned should significantly contribute to our understanding of many epithelial cancers.

**Acknowledgements**
This work was supported in part by the Medical Research Council.

### References

CAIRNS P, TOKINO K, EBY Y AND SIDRANSKY D. (1994). Homozygous deletions of 9p21 in primary human bladder tumours detected by comparative multiplex polymerase chain reaction. *Cancer Res.*, **54**, 1422–1424.

CAIRNS P, TOKINO K, EBY Y AND SIDRANSKY D. (1995). Localisation of tumor suppressor loci on chromosome 9 in primary human renal cell carcinomas. *Cancer Res.*, **55**, 224–227.

CARBONARA C, LONGA L, GROSSO E, BORRONE C, GRAZIA-GARRE M, BRISIGOTTI M AND MIGONE N. (1994). 9q34 loss of heterozygosity in a tuberous sclerosis astrocytoma suggests a growth suppressor-like activity also for the TSC1 gene. *Hum. Mol. Genet.*, **3**, 1829–1832.

CHENEVIX-TRENCH G, KERR J, FRIEDLANDER M, HURST T, SANDERSON B, COGLAN M, WARD B, LEARY J AND KHOO S-K. (1994). Homozygous deletions on the short arm of chromosome 9 in ovarian adenocarcinoma cell lines and loss of heterozygosity in sporadic tumors. *Am. J. Hum. Genet.*, **55**, 143–149.

CLIBY W, RITLAND S, HARTMANN L, DOBSON M, HALLING KC, KEENEY G, PODRATZ KC AND JENKINS RB. (1993). Human epithelial ovarian cancer allelotype. *Cancer Res.*, **53**, 2393–2398.

DEVLIN J, KEEN AJ AND KNOWLES MA. (1994). Homozygous deletion mapping at 9p21 in bladder carcinoma defines a critical region within 2 cM of IFNA. *Oncogene*, **9**, 2757–2760.

ECCLES D, CRANSTON G, STEEL CM, NAKAMURA Y AND LEONARD RCF. (1990). Allelelosses on chromsome 17 in human epithelial cancer. *Oncogene*, **5**, 1599–1601.

FOUNTAIN JW, KARAYIORGOU M, ERNSTOFF MS, KIRKWOOD JM, VLOCK DR, TITUS-ERNSTOFF L, BOUCHARD B, VIJAYA-SARDHI S AND DRACOPOLI NC. (1992). Homozygous deletions within human chromosome band 9p21 in melanoma. *Proc. Natl Acad. Sci. USA*, **89**, 10557–10561.

HABUCI T, DEVLIN J, ELDER PA AND KNOWLES MA. (1995). Detailed deletion mapping of chromosome 9q in bladder cancer: evidence for two tumour suppressor loci. *Oncogene*, **11**, 1671–1674.

KAMB A, GRUIS NA, WEAVER-FELDHAUS J, LIU Q, HARSHMAN K, TAVTIGIAN SV, STOCKERT E, DAY RS, JOHNSON BE AND SKOLNICK MH. (1994). A cell cycle regulator potentially involved in genesis of many tumor types. *Science*, **264**, 436–440.

KEEN AJ AND KNOWLES MA. (1994). Definition of two regions of deletion on chromosome 9 in carcinoma of the bladder. *Oncogene*, **9**, 2083–2088.

MERLO A, GABRIELSON E, MABRY M, VOLLMER R, BAYLIN SB AND SIDRANSKY D. (1994). Homozygous deletion on chromsome 9p and loss of heterozygosity on 9q, 6p and 6q in primary human small cell lung cancer. *Cancer Res.*, **54**, 2322–2326.

NOBORI T, MIURA K, WU DJ, LOIS A, TAKABAYASHI K AND CARSON DA. (1994). Deletions of the cyclin-dependent kinase-4 inhibitor gene in multiple human cancers. *Nature*, **368**, 753–756.

OSBORNE RJ AND LEECH V. (1994*a*). Polymerase chain reaction allelotyping of human ovarian cancer. *Br. J. Cancer*, **69**, 429–438.

OSBORNE RJ AND LEECH V. (1994*b*). Chromosome 9q deletion mapping in epithelial ovarian cancer. *Proc. Am. Assoc. Cancer Research*, **35**, 601.

POVEY S, ARMOUR J, FARNDON P, HAINES JL, KNOWLES MA, OLOPADE F, PILZ A, WHITE J, MEMBERS OF THE UTAH GENOME CENTER GENETIC MARKER AND MAPPING GROUP AND KWIATKOWSKI DJ. (1994). Report on the third international workshop on chromosome 9. *Ann. Hum. Genet.*, **58**, 177–250.

QUINN AG, SIKKINK S AND REES JL. (1994). Delineation of two distinct deleted regions on chromosome 9 in human non-melanoma skin cancers. *Genes, Chrom. Cancer*, **11**, 222–225.

SCHULTZ DC, VANDERVEER L, BUETOW KH, BOENTE MP, OZOLS RF, HAMILTON TC AND GODWIN AK. (1995). Characterisation of chromosome 9 in human ovarian neoplasia identifies frequent genetic imbalance on 9q and rare alterations involving 9p, including *CDKN2*. *Cancer Res.*, **55**, 2150–2157.

SHANLEY SM, DAWKINS H, WAINWRIGHT BJ, WICKING C, HEENAN P, ELDON M, SEARLE J AND CHENEVIX-TRENCH G. (1995). Fine deletion mapping on the long arm of chromosome 9 in sporadic and familial basal cell carcinomas. *Hum. Mol. Genet.*, **4**, 129–133.

VAN DER RIET P, NAWROZ H, HRUBAN RH, CORIO R, TOKINO K, KOCH W AND SIDRANSKY D. (1994). Frequent loss of chromosome 9p21–22 early in head and neck cancer progression. *Cancer Res.*, **54**, 1156–1158.

WILLIAMSON MP, ELDER PA, SHAW ME, DEVLIN J AND KNOWLES MA. (1995). p16 (CDKN2) is a major deletion target at 9p21 in bladder cancer. *Hum. Mol. Genet.*, **4**, 1569–1577.